# The Effect of Intravenous Albumin Administration Before Exchange Transfusion in Infants With Hyperbilirubinemia: A Meta‐Analysis

**DOI:** 10.1002/hsr2.71079

**Published:** 2025-07-27

**Authors:** Romina Kamyab, Siroos Hemmatpour, Ramyar Rahimi Darehbagh, Yousef Moradi, Nona Amirimanesh

**Affiliations:** ^1^ School of Medicine, Besat Hospital Kurdistan University of Medical Sciences Sanandaj Iran; ^2^ Department of Pediatrics, School of Medicine, Besat Hospital Kurdistan University of Medical Sciences Sanandaj Iran; ^3^ Student Research Committee Kurdistan University of Medical Sciences Sanandaj Iran; ^4^ Nanoclub Elites Association Tehran Iran; ^5^ Cellular and Molecular Research Center, Research Institute for Health Development Kurdistan University of Medical Sciences Sanandaj Iran; ^6^ Universal Scientific Education and Research Network (USERN) Sanandaj Kurdistan Iran; ^7^ Social Determinant of the Health Research Center, Research Institute for Health Development Kurdistan University of Medical Sciences Sanandaj Iran; ^8^ Department of Neonatology Shahid Beheshti University of Medical Sciences (SBUMS) Tehran Iran

**Keywords:** bilirubin, hyperbilirubinemia, intravenous albumin administration, meta‐analysis, phototherapy

## Abstract

**Background and Aims:**

This meta‐analysis aimed to assess the impact of intravenous albumin administration before exchange transfusion in infants diagnosed with hyperbilirubinemia.

**Methods:**

A comprehensive search was conducted in multiple electronic databases, including PubMed (Medline), Scopus, Embase, Web of Science, Cochrane Library, and ClinicalTrials.gov, using relevant keywords. The quality of the included randomized controlled trials (RCTs) was evaluated using the Cochrane Risk of Bias tool. Statistical analyses were performed using STATA (Version 18) and RevMan (Version 5).

**Results:**

Four RCTs met the inclusion criteria. Pooled analysis indicated a nonsignificant reduction in total bilirubin levels following intravenous albumin administration (SMD: −2.19; 95% CI: –4.79 to 0.41; *I*² = 68.03%; *p* for heterogeneity = 0.001). Subgroup analysis revealed no significant reduction in early bilirubin measurements (≤ 6 h), but a statistically significant reduction was observed in later measurements (12–24 h) (SMD: −2.91; 95% CI: –4.93 to –0.89). The duration of phototherapy was significantly reduced in the albumin group compared to placebo (WMD: –11.23; 95% CI: –21.28 to –1.19; *I*² = 76.12%; *p* for heterogeneity = 0.03).

**Conclusion:**

While intravenous albumin administration before exchange transfusion did not show a statistically significant effect on total bilirubin levels overall, a potential bilirubin‐lowering effect was observed when measured at later time points. Moreover, albumin significantly reduced the duration of phototherapy. These findings suggest potential clinical benefit, though further high‐quality RCTs are warranted.

## 1 Introduction

Neonatal jaundice is a prevalent condition that frequently leads to hospital readmission in infants [1]. It manifests as a yellowish discoloration of the skin, mucous membranes, and other tissues due to elevated bilirubin levels [2]. Hyperbilirubinemia results from an imbalance between bilirubin production and conjugation, leading to increased total serum bilirubin (TSB) concentrations. This condition is typically diagnosed when bilirubin levels exceed 171 µmol/L in preterm infants or 256 µmol/L in full‐term neonates [2, 3].

Several factors contribute to hyperbilirubinemia, including the immaturity of hepatic enzymes, rapid breakdown of red blood cells, and increased enterohepatic circulation [4, 5, 6]. Genetic conditions such as glucose‐6‐phosphate dehydrogenase (G6PD) deficiency and Gilbert's syndrome also elevate the risk of severe neonatal hyperbilirubinemia by impairing bilirubin metabolism [7, 8].

Neonatal jaundice is observed in approximately 60%–80% of preterm and full‐term infants, with a higher prevalence among exclusively breastfed neonates [9, 10]. If left untreated, severe hyperbilirubinemia can result in significant complications, including bilirubin‐induced neurological dysfunction, acute bilirubin encephalopathy, and kernicterus [11]. Global estimates indicate that at least 481,000 infants develop severe hyperbilirubinemia annually, with over 130,000 at risk of bilirubin‐induced brain injury or mortality [12, 13]. Among affected infants, more than 63,000 may experience long‐term neurological impairment [11, 13, 14].

Effective management of neonatal jaundice is crucial to prevent bilirubin‐induced neurotoxicity [15]. The American Academy of Pediatrics has established guidelines for managing neonatal jaundice, including standard phototherapy, intensive phototherapy, albumin infusion, and, in severe cases, exchange transfusion [16]. Phototherapy is widely accepted as a first‐line treatment due to its noninvasive nature and effectiveness [17]. However, prolonged phototherapy may lead to complications such as dehydration, electrolyte imbalances, and circadian rhythm disturbances [18]. In cases where phototherapy fails to adequately control bilirubin levels, exchange transfusion is performed to prevent neurotoxicity [19].

Albumin infusion has been proposed as an adjunct therapy before exchange transfusion to enhance bilirubin clearance [20]. Albumin binds to circulating bilirubin, increasing its solubility and facilitating excretion. Several studies have explored the effects of administering albumin before exchange transfusion, yielding mixed results. Some studies report a significant reduction in post‐exchange bilirubin levels and a shorter duration of phototherapy, while others have found no significant difference compared to standard treatment [20, 21, 22, 23].

Given the high cost and potential risks associated with albumin infusion, including fluid overload and infection, its routine use remains controversial. This meta‐analysis aims to systematically evaluate the effect of intravenous albumin administration before exchange transfusion on bilirubin levels and treatment outcomes in infants with hyperbilirubinemia.

## 2 Methods

This systematic review and meta‐analysis was conducted in accordance with the Preferred Reporting Items for Systematic Reviews and Meta‐Analyses (PRISMA) 2020 guidelines [24]. The study protocol was prospectively registered in PROSPERO (CRD42023422185).

### 2.1 Search Strategy

A comprehensive literature search was performed across the following databases: PubMed (Medline), Scopus, Embase, Web of Science, Cochrane Library, and ClinicalTrials.gov, covering studies published between January 1990 and December 2024. The search strategy was designed using Boolean operators (AND, OR) with key terms including “Neonatal Hyperbilirubinemia”, “Bilirubin”, “Phototherapy”, “Hospital Stay”, and “Albumin Administration”. Synonyms were identified using MeSH, Emtree, and Thesaurus terms (Table 1). To ensure comprehensive coverage, manual searching of reference lists from relevant studies was also performed.

**Table 1:** Search terms, databases, and inclusion/exclusion criteria.

Category	Details
Search Terms	“Neonatal Hyperbilirubinemia”, “Bilirubin”, “Phototherapy”, “Hospital Stay”, “Albumin Administration” Synonyms identified using MeSH, Emtree, and Thesaurus terms.
Databases Searched	PubMed (Medline), Scopus, Embase, Web of Science, Cochrane Library, ClinicalTrials.gov
Inclusion Criteria	**Population:** Infants with hyperbilirubinemia **Intervention:** Albumin administration before plasma exchange **Comparison:** Placebo or other routine treatments **Outcomes:** Phototherapy duration and bilirubin levels **Study Design:** Randomized controlled trials (RCTs)
Exclusion Criteria	Duplicate publications, review articles, cross‐sectional studies, case‐control or cohort studies, books, conference abstracts, and clinical trials with different primary interventions or outcomes.

### 2.2 Eligibility Criteria

Studies were selected based on the Population, Intervention, Comparison, Outcome, and Study design (PICOS) framework (Table 1). The population of interest was infants with hyperbilirubinemia, and the intervention being investigated was albumin administration before exchange transfusion. The comparison group included studies using placebo or other routine treatments, while the outcomes measured were phototherapy duration and bilirubin levels. Only randomized controlled trials (RCTs) were included, as they are considered the gold standard for clinical evidence (Table 1).

### 2.3 Study Selection and Data Extraction

All retrieved studies were imported into EndNote (Version 8) for deduplication. Two independent reviewers screened studies at the title, abstract, and full‐text levels, with discrepancies resolved through discussion or consultation with a third reviewer. Data were extracted independently by two reviewers and included study characteristics (authors, year, country, study design, sample size), patient characteristics (age, sex, birth weight), intervention details (type, dose, and duration of albumin administration), comparison group details, and outcomes (hospitalization duration, phototherapy duration, bilirubin levels).

### 2.4 Risk of Bias Assessment

The risk of bias in the included RCTs was assessed using Version 2 of the Cochrane Risk of Bias tool (RoB 2) [25]. Two reviewers independently assessed each study, with disagreements resolved by discussion.

### 2.5 Statistical Analysis

The meta‐analysis was conducted using STATA software (Version 18). Either Standardized Mean Difference (SMD) or Weighted Mean Difference (WMD) was calculated for continuous outcomes, depending on data availability. SMD was applied when means and standard deviations were reported both before and after intervention, while WMD was used for post‐intervention data only. For studies reporting medians and ranges, means and standard deviations were estimated using the Hozo et al. (2005) [26] method. The timing of measurements in each study was thoughtfully chosen, selecting the most clinically relevant timepoint when multiple measurements were available. This approach ensured that each study contributed only once per endpoint (end time). Effect sizes were pooled using a random‐effect model, with heterogeneity assessed using Cochrane's *Q* test and *I*² statistic.

Publication bias was evaluated using Egger's test, and subgroup analyses were performed by birth weight and albumin dose. Risk of bias was assessed using RevMan software (Version 5.0) following Cochrane guidelines. All analyses maintained a significance threshold of *p* < 0.05. Subgroup analyses were performed based on birth weight and different doses of albumin. The significance level in this meta‐analysis was considered below 0.05. To account for variation in the timing of bilirubin measurements across studies, a subgroup analysis was performed based on the time point of outcome assessment following exchange transfusion. Studies were categorized into two subgroups: early measurements, defined as total serum bilirubin levels assessed within ≤ 6 h, and late measurements, defined as bilirubin levels measured between 12‐ and 24‐h post‐transfusion. If a study reported data for more than one time point, it was included in each relevant subgroup. Specifically, the study by Shahian et al. [27] was included twice (once in the early subgroup (6 h) and again in the late subgroup (24 h) to reflect the multiple time points reported in that trial.

## 3 Results

**Figure 1:**
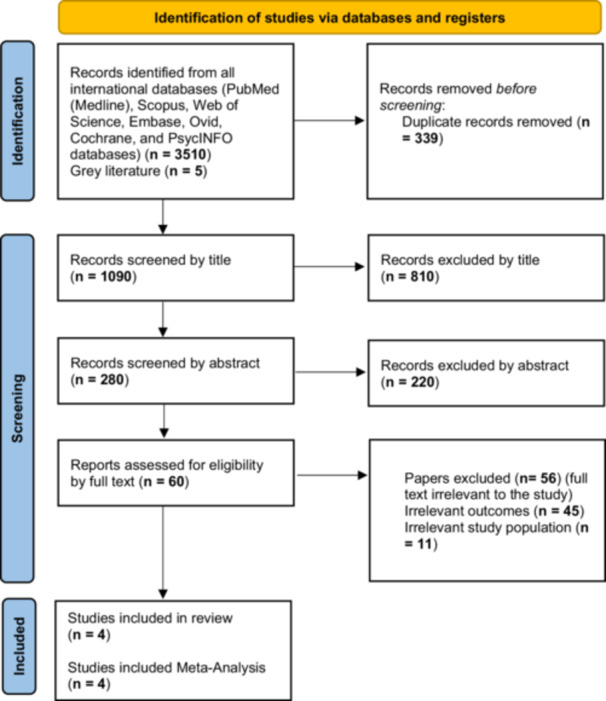
PRISMA 2020 flow diagram for new systematic reviews which included searches of databases and registers only.

In this meta‐analysis, after the search strategy and screening of articles based on the topic, abstract and full text, finally, four studies remained for meta‐analysis based on the inclusion and exclusion criteria (Figure 1). Of the four selected clinical trial studies, three studies were conducted in Asia, one study was in Canada, one study was in Kenya, and the last one was in England. Among these studies, one study used 5% albumin, and the rest used 20% albumin for intervention. The overall average weight of infants in all studies was 2570 ± 354.48 g. In all of the included RCTs, one dose of albumin based on one kilogram of the baby's weight was considered for the intervention, also the time range of outcome measurement was from the time immediately after the intervention to 24 h later (Table 2).

**Table 2:** The characteristics of included studies.

Authors (Year)	Country	Sample Size (I/C)	Age (Days) Mean ± SD	Birth Weight (g) Mean ± SD	Gender (M/F)	Pre‐exchange TBIL (mg/dL)	Post‐exchange TBIL (mg/dL)	Intervention	Comparison	Measurement Time	Study Population
Shahian et al. (2006)	Iran	25/25	I: 7 ± 1.1 C: 8 ± 1.0	I: 3239 ± 585 C: 3264 ± 428	I: 13/12 C: 12/13	I6h: 14.4 ± 1.7I12h: 8 ± 1.5I24h: 3.5 ± 0.5	Not reported	IV 20% albumin, 1 g/kg, within 1 h before ET	Standard ET	6 h and 24 h after ET (early & late)	Term neonates, GA > 37 weeks, BW > 2500 g, TSB > 25
Comley et al. (1967)	England	I1/I2/C: 17/17/19	Not reported	I1: 3.03 kgI2: 2.92 kgC: 2.96 kg	Not reported	I1h: 4.96 ± 0.67I4h: 3.37 ± 0.69	I1: 3.68 ± 0.86I2: 3.75 ± 0.51	I1: Albumin substitution during ETI2: IV albumin 2.5–10 g, 1–6 h before ET	Control (ET only)	Immediate & 6 h after ET (early)	Neonates > 10 h old, TBIL > 12 mg/dL
Dash et al. (2011–13)	India	23/27	I: 23.8 ± 3.2 h	I: 2952 gC: 2926 g	Not reported	I6h: 10.55 ± 1.53I12h: 5.86 ± 1.21	I: 19.0 ± 3.85 C: 24.8 ± 3.9	IV 5% albumin, 1 g/kg over 2 h, 2 h before ET	Maintenance IV fluid 20 mL/kg over 2 h	24 h after ET (late)	Term and late preterm, nonhaemolytic hyperbilirubinemia
Mitra et al. (2008–09)	India	21/21	I: 6.23 ± 1.6 C: 6.67 ± 1.43	I: 1619 ± 324 C: 1660 ± 320	I: 10/11 C: 12/9	C6h: 15.26 ± 1.78C12h: 11.69 ± 1.52	I: 11.9 ± 3.9 C: 12.4 ± 6.6	IV 20% albumin, 1 g/kg over 1 h before ET	0.9% saline 5 mL/kg over 1 h pre‐ET	24 h after ET (late)	Neonates, BW 1000–2499 g, GA ≥ 32 weeks

**Figure 2:**
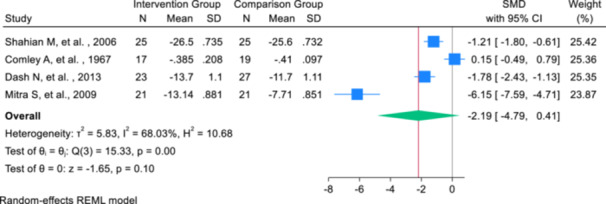
Forest plot of the change in total bilirubin levels (mg/dL) following intravenous albumin administration and exchange transfusion.

The primary outcome of this meta‐analysis was to determine the SMD of total bilirubin levels. The sample size consisted of 86 infants in the intervention group and 92 infants in the comparison group. The meta‐analysis results indicated that the use of intravenous albumin administration before exchange transfusion in infants with hyperbilirubinemia resulted in a reduction of total bilirubin by 2.19 (SMD: ‐2.19; 95% CI: ‐4.79, 0.41; *I*²: 68.03%; *p*‐heterogeneity > 0.001), with no significant effect (Figure 2). The effect sizes from the selected studies ranged from −6.15 to 0.15, with the highest effect observed in the study by Mitra et al. [28] and the lowest in the study by Comley et al. [29] (Figure 2).

Meta‐regression analyses based on the infants' age and weight showed that, with increasing age (in days), the effect of albumin administration before exchange transfusion on reducing the total bilirubin level increased by 0.012. However, this increase was not statistically significant (*B*: 0.012; SE: 0.076; *p*‐value: 0.580; 95% CI: −0.013, 0.259). The effect based on the weight of infants (in grams) was negligible and also not statistically significant (*B*: −0.043; SE: 0.099; *p*‐value: 0.921; 95% CI: −0.079, 0.001). To assess publication bias, the Eggers test was conducted, as the number of studies included was fewer than 10. The results indicated a potential publication bias in the study of the effect of albumin before exchange transfusion on the average duration of phototherapy (*B*: −8.71; SE: 3.00; *p*‐value: 0.361).

Subgroup analyses were conducted based on the type and concentration of albumin administered, the weight of the neonates, and the publication year of the studies. Regarding the type of intervention, albumin was administered intravenously at concentrations of either 5% or 20% before exchange transfusion. A subset of studies did not specify the albumin concentration but indicated that dosage was adjusted according to infant weight; these were grouped under the “others” category. Subgroup analysis revealed that infants receiving 5% albumin exhibited a significantly greater reduction in total bilirubin compared to placebo (SMD: –6.15; 95% CI: –7.59, –4.71), although this result was derived from a single study. In the 20% albumin group, the mean bilirubin level was also significantly lower (SMD: −1.48; 95% CI: −2.04, –0.92; *I*² = 35.56%), while the “others” group showed no significant effect (SMD: 0.15; 95% CI: −7.59, 0.79). The between‐group heterogeneity was statistically significant (*Q* = 63.31, *p* < 0.001), indicating differences in treatment effect by albumin type (Table 3).

**Table 3:** The effect of albumin administration on the total bilirubin in neonatal with hyperbilirubinemia based on type of administrations and neonatal weight.

Category	Subgroup	SMD (95% CI)	Within‐group heterogeneity (*Q*, *I*², *p*)	Between‐group heterogeneity (*Q*, *p*)
Type of administration	5% Human Albumin	−6.15 (−7.59, −4.71)	Not reported	*Q* = 63.31, *p* = 0.001
	20% Human Albumin	−1.48 (−2.04, −0.92)	*Q* = 1.63, *I*² = 35.56%, *p* = 0.200	
	Others	0.15 (−7.59, 0.79)	Not reported	
Weight	≤ 2500 g	−2.96 (−9.14, 3.21)	*Q* = 18.00, *I*² = 72.37%, *p* = 0.001	*Q* = 0.22, *p* = 0.641
	> 2500 g	−1.48 (−2.04, −0.92)	*Q* = 1.63, *I*² = 38.56%, *p* = 0.200	
Publication year	Before 2000	0.15 (−0.49, 0.79)	Not reported	*Q* = 8.04, *p* = 0.001
	After 2000	−2.91 (−4.93, −0.89)	*Q* = 28.64, *I*² = 84.81%, *p* = 0.001	
Time of measurement	Early (≤ 6 h)	−4.46 (−13.60, 4.67)	*Q* = 18.88, *I*² = 77.51%, *p* = 0.001	*Q* = 0.08, *p* = 0.963
	Late (12–24 h)	−2.91 (−4.93, −0.89)	*Q* = 16.93, *I*² = 69.40%, *p* = 0.001	

Infants were also categorized by weight: ≤ 2500 g or > 2500 g. In those weighing ≤ 2500 g, albumin administration resulted in a nonsignificant bilirubin reduction (SMD: −2.96; 95% CI: –9.14, 3.21; *I*² = 72.37%), while a significant effect was observed in those > 2500 g (SMD: –1.48; 95% CI: –2.04, –0.92; *I*² = 38.56%). When studies were stratified by publication year, those published after 2000 demonstrated a significant reduction in bilirubin (SMD: –2.91; 95% CI: –4.93, –0.89; *I*² = 84.81%), whereas studies published before 2000 showed no significant effect (SMD: 0.15; 95% CI: –0.49, 0.79). Heterogeneity was higher in post‐2000 studies. These findings suggest that treatment effect may vary with albumin concentration, infant weight, and study era (Table 3).

The subgroup analysis based on timing of bilirubin measurement showed differing effects. In the early measurement group ( ≤ 6 h), the pooled SMD was –4.46 (95% CI: –13.60 to 4.67; *p* = 0.963; *I*² = 77.51%), suggesting no significant effect and considerable heterogeneity. In contrast, the late measurement group (12–24 h) showed a statistically significant reduction in bilirubin levels (SMD: –2.91; 95% CI: –4.93 to –0.89; *p* < 0.001; *I*² = 69.40%). It is important to note that the study by Shahian et al. was included in both the early and late subgroups, as it provided data at two time points: 6 h and 24 h post‐transfusion (Table 3).

**Figure 3:**
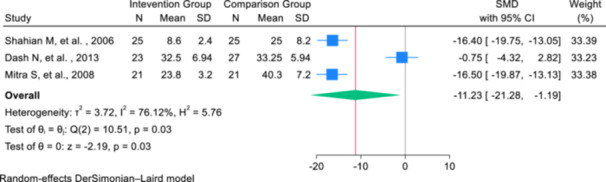
Forest plot of the effect of albumin administration on the phototherapy time in neonatal with hyperbilirubinemia.

The secondary outcome of this meta‐analysis was the determination of the WMD in the duration of phototherapy between the intervention and placebo groups. Since the baseline duration of phototherapy was not reported in the selected studies, the weighted mean difference was calculated by reporting the standardized mean difference for the change from baseline to post‐intervention in each group. Three studies provided data on the average duration of phototherapy after the intervention. The meta‐analysis included three studies comparing the effect of intravenous albumin administration before exchange transfusion to a placebo. The combined SMD for the primary outcome was −11.23 (95% CI: −21.28, −1.19), indicating a significant reduction in the outcome favoring the albumin intervention. However, there was high heterogeneity among the studies, with an *I*² value of 76.12%, suggesting considerable variability in the results. The *Q*‐test for homogeneity was significant (*Q* = 10.51, *p* = 0.031), reinforcing the presence of substantial heterogeneity (Figure 3).

Meta regression analyses were performed based on the age and weight of the infants, and the results showed that with the increase of infants' age (based on days), the effect of albumin before exchange transfusion on the mean duration of phototherapy increases by 0.023 (*B*: 0.023; SE: 0.003; *p* value: 0.0001; 95% CI: 0.016, 0.030). Also, this effect increases slightly based on the weight of infants (in grams), but it is not statistically significant (*B*: 0.007; SE: 0.001; *p* value: 0.645; 95% CI: −0.001, 0.008). To check the publication bias, since the number of articles was less than 10, only the Eggers test results were reported, and based on these results, publication bias occurred in the results of investigating the effect of albumin before exchange transfusion on the average duration of phototherapy *B*: −18.97; SE: 4.52; *p* value: 0.0001).

**Figure 4:**
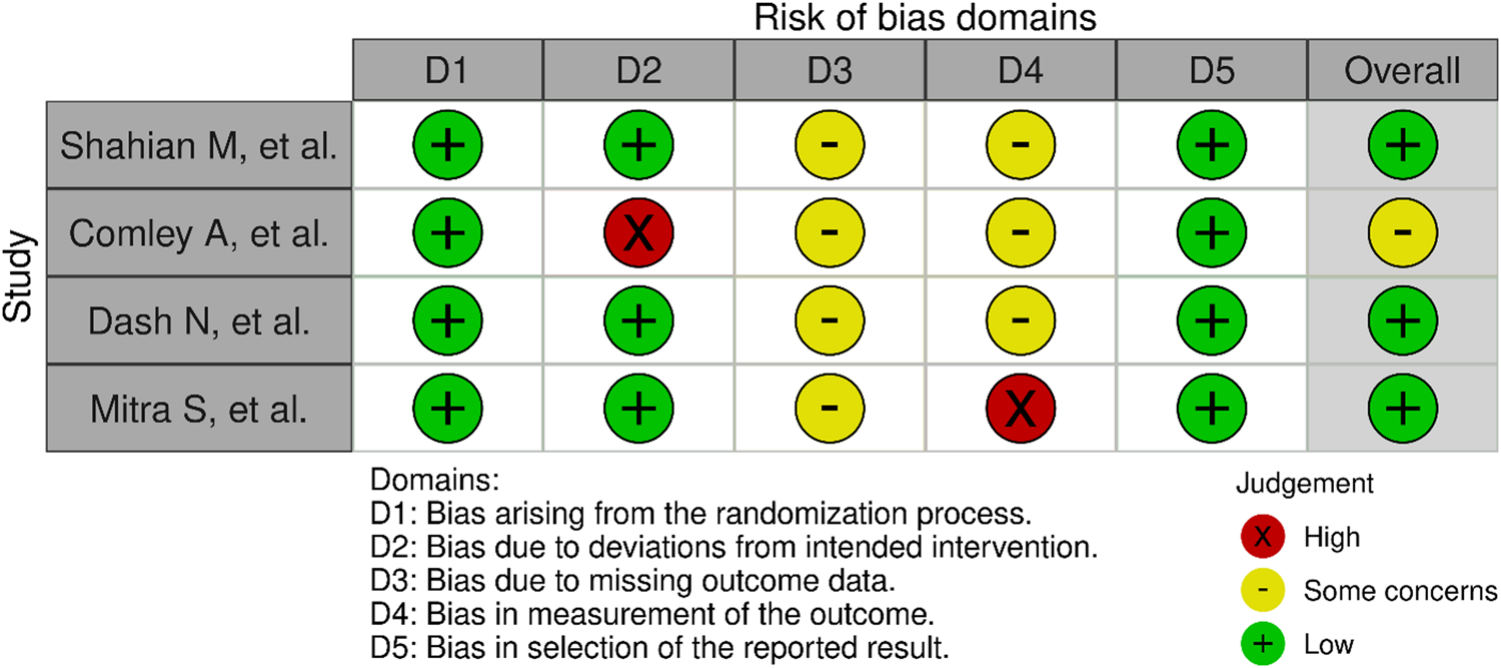
Risk of bias graph and Risk of bias summary for each included study.

The quality assessment analyses conducted in this meta‐analysis indicated that all included studies were classified as having a low risk of bias in terms of random sequence generation and other sources of bias, suggesting that appropriate randomization methods were applied. However, the highest proportion of studies categorized as having an unclear risk of bias was observed in the domains of blinding of participants and personnel and blinding of outcome assessment, likely due to insufficient methodological details or a lack of clear reporting. Conversely, the risk of bias related to allocation concealment, incomplete outcome data, and selective reporting was generally acceptable, with most studies demonstrating adequate methodological rigor in these aspects. Nonetheless, caution is warranted when interpreting the findings, as potential biases in blinding may impact the overall reliability of the results (Figure 4).

## 4 Discussion

This meta‐analysis evaluated the effectiveness of albumin infusion before blood exchange in managing neonatal hyperbilirubinemia. Four clinical trials were reviewed, revealing that albumin infusion before exchange transfusion reduces total bilirubin levels and shortens the duration of phototherapy; however, only the results related to phototherapy were statistically significant.

Hyperbilirubinemia, if untreated, can lead to severe neurological complications, including bilirubin‐induced encephalopathy, due to unconjugated bilirubin (UCB) crossing the blood‐brain barrier and causing brain damage through apoptosis, necrosis, kernicterus, and, in extreme cases, death [30, 31, 32, 33]. Thus, timely interventions such as albumin infusion are critical for preventing such damage. Albumin has a well‐established role in binding unconjugated bilirubin, aiding in its removal from the body. As unconjugated bilirubin is soluble in water at physiological pH, it requires binding to albumin for efficient metabolism in the liver and excretion [34, 35, 36]. The results of this meta‐analysis support the hypothesis that increasing the binding capacity of bilirubin by administering albumin intravenously enhances bilirubin clearance, thus reducing its potential for central nervous system toxicity. This mechanism is particularly important in preventing brain injury in neonates with hyperbilirubinemia. Our findings confirm that intravenous albumin infusion before exchange transfusion is an effective treatment for neonatal hyperbilirubinemia, contributing to both bilirubin reduction and shortened phototherapy duration.

Additionally, albumin infusion in the intravascular space promotes the movement of tissue‐bound bilirubin into the plasma, which facilitates its removal during exchange transfusion. This action further reduces the total serum bilirubin levels and prevents a rebound increase after exchange [37, 38]. Albumin injection before exchange transfusion leads to more effective removal of intravascular unconjugated bilirubin, subsequent reduction of total serum bilirubin, and reduction in the return of unconjugated bilirubin to its previous value after exchange transfusion [39, 40].

Notably, subgroup analysis revealed that 5% albumin was more effective than 20% albumin in lowering bilirubin levels. Infants who received 5% albumin experienced a substantial reduction of 6.15 in total bilirubin, while those treated with 20% albumin showed a more modest effect. This finding suggests that a lower albumin concentration may provide a more favorable binding environment, enhancing bilirubin clearance. Nevertheless, further research is warranted to determine the optimal concentration, dosage, and administration protocols to maximize therapeutic outcomes. Weight‐based subgroup analysis indicated a greater bilirubin reduction in infants weighing ≤ 2500 g (SMD: –2.96) compared to those over 2500 g (SMD: −1.48). Although the mechanisms underlying this differential response remain uncertain, it is plausible that differences in body composition, albumin distribution, or bilirubin metabolism may influence treatment efficacy in lower birth weight neonates. These findings highlight the need for more targeted investigations into how infant weight modifies treatment response. In addition, a marked disparity was observed between studies published before and after 2000. Studies conducted after 2000 reported significantly stronger and more consistent effects, likely due to improved methodological rigor, standardized dosing protocols, and greater attention to weight‐adjusted dosing. In contrast, older studies were often limited by inconsistent dosing strategies and greater methodological heterogeneity, contributing to wider confidence intervals and increased uncertainty in effect estimates. These temporal trends emphasize the importance of methodological refinement in clinical research and its impact on the reliability of treatment effect estimates. The analysis of bilirubin levels stratified by measurement time points revealed a potential time‐dependent treatment effect of albumin. No significant effect was observed in the early subgroup, possibly due to ongoing redistribution of bilirubin immediately after exchange transfusion. Conversely, a significant reduction in bilirubin levels was detected in the late subgroup, supporting the hypothesis that albumin's bilirubin‐binding effects may become more apparent over time. The inclusion of Shahian et al.'s study [27] in both subgroups, reflecting its dual time point measurements (6 and 24 h), provided an opportunity to evaluate this dynamic effect more accurately. This approach underscores the value of multi‐timepoint reporting and the need for standardized outcome measurement intervals in future trials The reduction in phototherapy duration by 1.87 days in the albumin‐treated group underscores the clinical utility of albumin infusion in managing hyperbilirubinemia. The effect of albumin on phototherapy duration was found to increase slightly with the age of the infant, although the increase was minimal and statistically insignificant for weight. These findings align with previous studies suggesting that older infants may exhibit a more robust response to treatment [18]. Although a higher level of serum albumin may provide more binding sites for bilirubin and may facilitate better diffusion of bilirubin into the intravascular space, how should albumin concentration be determined or whether higher albumin concentrations for infants with their baseline serum albumin is low, whether it should be used or not, and also determining the amount of albumin concentration according to the baby's weight needs to be evaluated. Therefore, the results of bilirubin reduction following exchange transfusion require more clinical trials.

The present meta‐analysis is the first meta‐analysis in the world that has been comprehensively conducted to determine the effect of intravenous albumin administration before exchange transfusion in infants with hyperbilirubinemia. The results of the present meta‐analysis can be very effective in completing and updating clinical guidelines in the field of infants and treating hyperbilirubinemia. One of the limitations of this study was the lack of subgroup analysis based on other important and other influencing variables on hyperbilirubinemia and the lack of reporting of other outcomes, the main reason for which was the lack of reporting of these variables in the initial studies. In addition, due to the small size of the samples in the selected clinical trial studies, the sample size in this meta‐analysis was low. So, it is suggested that future clinical trial studies consider a larger sample size and report other important outcomes related to hyperbilirubinemia.

## 5 Conclusion

This meta‐analysis demonstrates that administering albumin before exchange transfusion significantly reduces total serum bilirubin levels and the duration of phototherapy in neonates with hyperbilirubinemia. This intervention may help shorten hospitalization time and reduce associated healthcare costs for families. Furthermore, these findings offer valuable evidence that could inform updates to clinical guidelines and support more effective, evidence‐based decision‐making in the management of neonatal hyperbilirubinemia.

## Author Contributions

**Romina Kamyab:** data curation, investigation, validation, formal analysis, supervision, writing – original draft, writing – review and editing, resources. **Siroos Hemmatpour:** conceptualization, methodology, formal analysis, supervision, validation, investigation, funding acquisition, writing – review and editing, writing – original draft. **Ramyar Rahimi Darehbagh:** writing – review and editing, writing – original draft. **Yousef Moradi:** writing – original draft, writing – review and editing, software, methodology. **Nona Amirimanesh:** writing – original draft, writing – review and editing, conceptualization, methodology, software, data curation, supervision, formal analysis, validation, investigation, funding acquisition, project administration, resources.

## Conflicts of Interest

The authors declare no conflicts of interest.

## Transparency Statement

The lead author Nona Amirimanesh affirms that this manuscript is an honest, accurate, and transparent account of the study being reported; that no important aspects of the study have been omitted; and that any discrepancies from the study as planned (and, if relevant, registered) have been explained.

## Data Availability

The data that support the findings of this study are available from the corresponding author upon reasonable request.
